# Breast hibernoma in a male patient: a rare case report and review of the literature

**DOI:** 10.1093/jscr/rjad239

**Published:** 2023-05-12

**Authors:** Sam Hanna, Arie Davis, Jason Diab, Zackariah Clement

**Affiliations:** Tweed Hospital, Department of Surgery, New South Wales, Australia; John Flynn Private Hospital, Department of Surgery, Queensland, Australia; University of Notre Dame, School of Medicine, Sydney, Australia; Griffith University, School of Medicine, Queensland, Australia; Tweed Hospital, Department of Surgery, New South Wales, Australia; John Flynn Private Hospital, Department of Surgery, Queensland, Australia; University of Notre Dame, School of Medicine, Sydney, Australia; School of Medicine, University of New South Wales, Sydney, Australia; Tweed Hospital, Department of Surgery, New South Wales, Australia; John Flynn Private Hospital, Department of Surgery, Queensland, Australia

**Keywords:** Hibernoma, breast, mammary, brown fat

## Abstract

Hibernomas are uncommon, benign, lipomatous tumours of brown fat. Although hibernomas may arise from any region where brown fat exists, common locations include thigh, shoulder, back and neck. We report a rare finding of a breast hibernoma in a 43-year-old male. The patient was managed surgically with an excision of the breast mass. This report will outline the pathology and clinical findings of breast hibernomas and review of the literature.

## INTRODUCTION

Hibernomas are uncommon, benign, lipomatous tumours of brown fat [1]. Brown fat is mainly present during foetal life and replaced by white adipose tissue; however, remnants of brown fat can persist into adult life [2]. The peak incidence of hibernomas is reported to occur within the third decade of life [3]. They commonly occur in areas of the thigh, shoulder and back. In this case report, we present the first male presentation of a hibernoma in mammary tissue and a review of the literature.

## CASE REPORT

**Figure 1:**
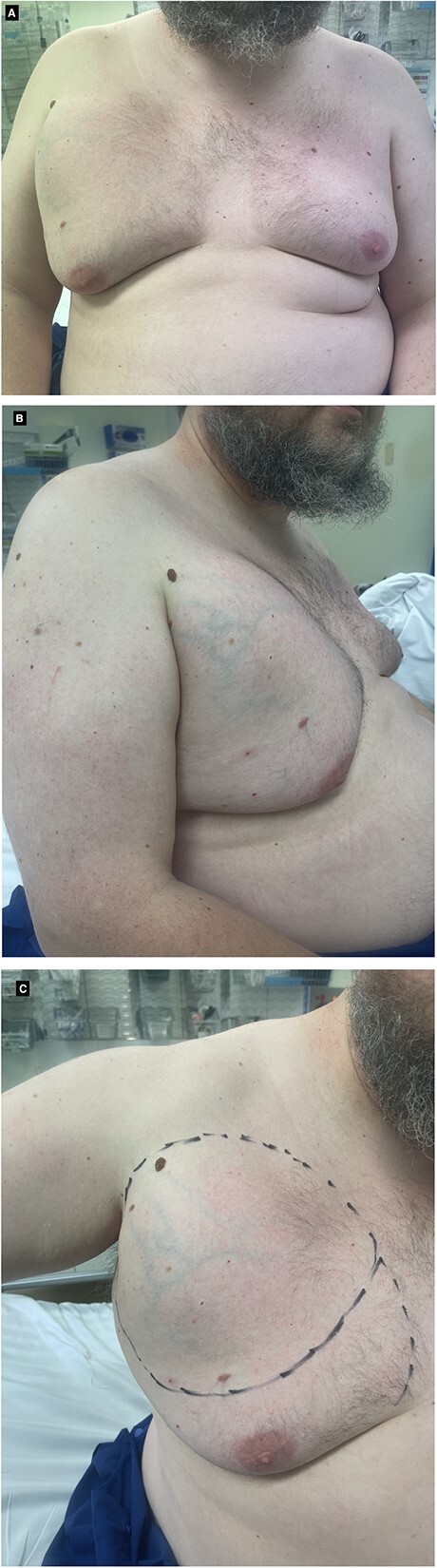
(**A**-**C**) Clinical photos of male breast hibernoma.

A 43-year-old male was referred by his General Practitioner to a breast surgeon with a large, right upper chest lump extending to his axilla. The lump had been present for almost a decade. Over the last several months, he had noticed it growing in size and discomfort with shoulder movement. He had no significant past medical history or family history of breast cancer or lipomas. He was a non-smoker and drinker with no regular medications. Clinically, there was a right-sided, upper-outer-quadrant breast mass, transitioning from firm to soft as it extended into the axilla. A small lump was also palpable at the 7 o’clock region without tenderness. The left breast demonstrated no tenderness or lumps, and no axillary or supraclavicular lymphadenopathy. The right nipple-areola-complex (NAC) was grade 3 ptosis and the left was grade 2, with bilateral soft gynecomastia present (Fig. 1).

He underwent an ultrasound, which revealed a large palpable lesion in the anterior right chest wall, measuring 16.3 × 5.4 × 16.1 cm, with a hyperechoic encapsulated lesion. The lesion contained a hypoechoic central focus measuring 2.1 cm without internal vascularity. At the 7 o’clock position, 6 cm from the nipple, there was a 26 × 17 mm hyperechoic avascular focus. He further underwent a breast magnetic resonance imaging (MRI), which demonstrated a circumscribed lesion of fat composition (68 × 130 × 140 mm) with mild internal vascularity, but no focal restricted diffusion or associated mass component. The lesion abutted the anterior margin of pectoralis major muscle and was away from the major neurovascular bundle. The patient underwent core biopsy of lesion, which confirmed the diagnosis of a hibernoma.

**Figure 2:**
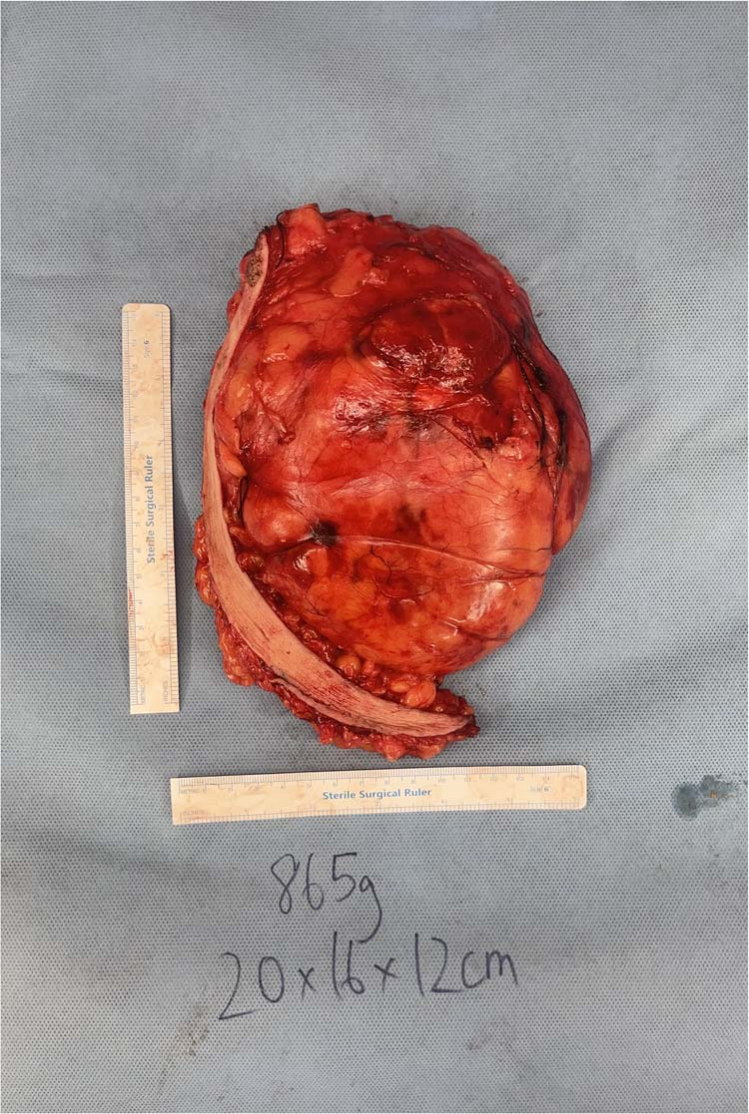
Intra-operative photos of breast hibernoma.

After extensive discussion, he underwent excision of right breast mass. Intra-operative findings included a large, partially encapsulated mass measuring 20 × 16 × 12 cm and weighing 865 g (Fig. 2). The mass was adherent to the pectoral fascia, but not infiltrating it and extending over lateral chest wall and axilla with extensive neovascularisation around the mass. Histopathological assessment showed a lobulated, fatty lesion with many of the adipocytes demonstrating features of brown fat and no evidence of malignancy. The patient recovered well post-operatively and was reviewed one month post-operatively with no complications.

## DISCUSSION

Hibernomas are lobulated, well circumscribed, partially encapsulated neoplasms with yellowish-brown, rubbery texture with an average size of 9 cm (range: 1–24 cm) [1]. There are four histological variants of hibernoma: typical (82%), myxoid (9%), lipoma-like (7%) and spindle-cell (2%) [1]. Within the typical subtype, the tumour consists of lobulated growth of multivacuolated pale, eosinophilic, and white fat cells in varying proportions in a background of rich capillaries [5]. At the ultrastructural level, the cells have characteristically many mitochondrias and well-defined lipid droplets packed within the cytoplasm [2, 4]. Immunohistochemical studies of hibernomas show global positivity for S100 protein in both eosinophilic and pale cells with variably intensity and negativity for CD34 [6, 7].

The largest series published to date about hibernomas (Soft Tissue Registry of the Armed Forces Institute of Pathology) reported 170 cases, of which 99 were men and 71 being women. The mean age of tumour development was 38.0 years ranging from 2 to 75 years [1]. Common anatomic locations included thigh, shoulder, back, neck, chest, arm, abdominal cavity, and retroperitoneum, accounting for only 1.6% of benign lipomatous neoplasms [6]. Breast hibernomas are relatively uncommon and almost all cases are reported in females. Clinically, majority of cases present as a slow-growing, asymptomatic mass, which can cause symptoms due to compression of adjacent structures or diagnosed as an incidental finding at a radiological routine examination [8].

A search of the literature was conducted using the terms ‘hibernoma’ and ‘breast’ or ‘mammary’ across multiple databases including Embase, Pubmed and Google Scholar. Relevant criteria included that the article was not a review article, included a diagnosis of hibernoma and was localized either to the breast or mammary tissue. Of the 495 articles identified, nine articles satisfied the criteria with an average age 45.5 years (range: 29–69). The most common presentation was a painless mass, occurring in five cases. Two cases reported pain, and the remainder two were incidental findings on routine imaging. The majority of cases were managed with surgical removal of the mass (7/9), with two patients opting for regular follow-up and reassurance. There were no cases of malignancy or neoplastic changes (Table 1).

**Table 1:** A literature review of case reports of breast hibernoma

**Author, year**	**Age** **sex**	**Clinical presentation**	**Imaging (US, CT, X-ray, MRI**	**Histopathology and immunochemistry**	**Management**	**Morbidity and recurrence**
**Dsouza, Cherian** [14]	68 F	Left breast: mass in upper quadrant for 1 month—rapidly increased in size over next 2 weeks Nil pain, discharge or skin changes OE—8 × 5 cm mass on examination—firm, irregular surface and restricted mobility Right breast—upper quadrant measuring 3 × 2 cm with irregular surface, consistency, and restricted mobility	US—encapsulated but irregular lesion in the left breast with increased vascularity	CNB—stromal fibrosis with no nuclear atypia or increased mitosis. Postop—diffuse areas of fibrosis and sheets of adipose cells showing fine multivacuolation of the cytoplasm interspersed with cells chowing univacuolation. Monovacuolated cells had eccentric nuclei. There was no nuclear atypia or increased mitosis with normal surrounding breast tissue.	Wide local excision of bilateral breast lumps	Postoperative period was noneventful Nil recurrence at 3 months
**Colville, Feigin** [15]	39 F	Asymptomatic—identified on screening mammography due to strong family breast cancer history	Mammogram—round, partially circumscribed partially indistinct, noncalcified 4.5 cm mass in the posterior one third of the upper outer quadrant of the right breast US—upper outer quadrant of right breast circumscribed, oval, revealed a well hyperechoic, parallel, septated mass measuring 6.2 × 4.7 cm	CNB—Lower power microscope = lobular growth pattern with multivacuolated immature fat cells admixed with univacuolated mature fat cells. High power microscopy—multivacuolated adipocytes with eosinophilic granular cytoplasm and numerous intercellular capillaries	Follow-up mammograms 6 months, 1 year and 2 years later demonstrating lesion stability	Nil
**Benn, Coetzee** [9]	45 F	8-month history of rapidly growing soft breast mass localised to the lower outer quadrant of the breast and chest wall with visibly distended veins and erythema. The mass was painful	Mammography—well-circumscribed opaque lesion, consistent with fat, and evidence of compressed surrounding tissue USS—Well defined mass of dense fat 130 mm in diameter	CNB—Unencapsulated proliferation of benign lipomatous elements resembling brown fat. The composite cells were large, round to ovoid granular eosinophilic cells with a distinct cellular membrane and multiple intracellular vacuoles containing multiple small lipid droplets. The nuclei were centrally placed, round and hyperchromatic with no nuclear atypia. Admixed, univacuolated mature adipocytes were also noted, with peripherally placed small, hyperchromatic, mature nuclei Postop—Multivacuolated brown fat cells with central nuclei. There was a mixture of pale cells alternating with eosinophilic cells and a delicate capillary network was noted between the brown fat cells	Excised through a lateral inframammary approach. Minimal dissection required.	Nil recurrence at 6 months follow up
**Neves Filho, Lima** [8]	42 F	6-month history of a painless mass in the upper outer quadrant of her right breast. Initial assessment showed a 10 cm x 10 cm lobulated, well-defined mobile mass. There was no associated lymphadenopathy	Mammography—A previous mammography revealed a regular, partially defined nodule in the upper outer quadrant of her right breast USS—Hypoechoic solid nodule of 10.2 × 5 cm, which occupied most of the upper outer and upper inner quadrants of the right breast	CNB—fibrous-adipose tissue associated with areas of steatonecrosis, without any evidence of neoplasia in those sample Postop—composed predominantly of lobules of large round to polygonal cells with abundant multivacuolated cytoplasm, well-defined membrane and central nuclei with fine chromatin and prominent nucleoli, which is consistent with a brown fat tumour, admixed with regular white adipocytes and small blood vessels. Immunochemistry—positivity for S100 protein, the tumour cells were negative for CD31, CD34, CD68 and topoisomerase	Partial mastectomy with marginal resection.	Nil
**Martini, Londero** [12]	42 F	Asymptomatic 42-year-old women with ovarian clear cell adenocarcinoma, treated at age 40 with chemotherapy and surgery, underwent CT-PET for staging. Abnormal uptake was noted in a focal captation area in the inferior inner quadrant of the right breast.	Mammography—Standard cardiocaudal and mediolateral oblique mammograms of the right breast did not demonstrate any abnormalities. There were no changes from routine mammograms two years prior. USS—Focused USS examination of the inferior inner quadrant of the right breast revealed a hyperechoic longitudinally orientated area, with a maximum diameter of 19 mm. MRI—precontrast T1 weighted images showed a partially ill-defined mass with signal intensity similar (slightly hypointense) to subcutaneous fat in the inferior inner quadrant of the right breast, measuring 18 × 18 × 31 mm. After rapid IV administration of gadolinium (0.1 mmol kg^−1^ Gd-DTPA followed by a 20 ml saline flush), the mass enhanced rapidly with a moderate enhancement rate (32% at 1 min 40 s) and a washout enhancement pattern. Repeat USS—examination confirmed the presence of a hyperechoic vascularised area in a well-defined hypoechoic fatty lobule, measuring 30 × 16 mm and corresponding to the MR finding.	CNB—Coarsely multivacuolated fat cells with small central nuclei and no atypia	Reassurance	Nil
**Gardner-Thorpe, Hirschowitz** [16]	29 F	1 month history of a lump in the upper outer quadrant of her left breast, breastfeeding her second child 3 months prior.	USS—partially well-defined, hyperechoic mass, 25 mm in diameter suggestive of post-lactational change. Graded benign (R2) and patient reassured. Repeat USS 3 months later with no change in size. Upgraded to R3 due to unusual sonographic appearance.	Postop—poorly defined nodule of brown fat. Cells arranged in lobules and had abundant, finely vacuolated cytoplasm with a small. Dark centrally placed nuclei.	Complete excision of a 25 × 25 × 20 mm soft yellow nodule	Nil
**Padilla-Rodriguez** [6]	37 F	Slow growing mobile painless mass in the upper-outer quadrant of her right mammary gland	USS—isoechoic nodular fatty mass. A 6-months previous screening mammogram and US showed no alterations	Postop—Tumour was composed to lobules of cells separated by thin fibrous septa intermixed with small vessels. Lobules were composed of predominately large round to polygonal cells with abundant multivacuolated pale cytoplasm, well-defined membrane, and central nuclei with fine chromatin and preominent nucleoli. Other cells presented with dense eosinophilic granular cytoplasm with scant univacuolated mature adipose cells at the periphery of the lesion Immunochemistry—strong positivity for S-100 protein and CD31 in both multivacuolated and eosinophilic cells. Negative for CD68 and CD34	Complete excision of a well-circumscribed lobulated soft nodule measuring 2.2 cm in its long axis	Nil
**Olthof, van Urk** [17]	62 F	Painful swelling in the right breast that was increasing in size.	USS and mammography—a sharply delineated hypervascular mass 14 cm in diameter with heterogeneous architecture. MRI—encapsulated swelling. After contract administration, multiple foci on dynamic images with accelerated enhancement and washout in the mass without axillary lymphadenopathy.	CNB was performed but inadequate sample was obtained	Extracapsular resection via incision in the inframammary fold with preventative drain placement	Post-operative bleeding required reoperation—a large clot was remove with correction of small, venous bleeding
**Runza, Blundo** [18]	F—age not known	Postmenopausal woman with a soft mass in the upper-outer quadrant of the left breast	Nil	4.3 cm lesion composed of abnormal population of monomorphic multivacuolated S-100 positive, CD-68 negative cells with no atypia that resembled normal brown adipose tissue	Surgical excision	Surgical margins were clean and patient is disease free at 23 months follow-up

The most common workup for breast masses follows the triple assessment with ultrasound, mammography and biopsy. Hibernomas on ultrasound present as hyperechoic, well-circumscribed ovular masses with uniform echogenicity [9], whereas angiography shows highly vascularized tumours with occasional arteriovenous shunts [10]. On MRI, lesions demonstrate signal intensity like subcutaneous fat on both T_1_ and T_2_ weighted images, with contrast enhancement due to hypervascularization [11]. However, no specific imaging features exist to distinguish hibernomas from other benign or malignant soft-tissue tumours [12], such as lipoma, angiolipoma, malignant fibrous histiocytoma and liposarcoma [13].

The present case was managed with complete surgical excision, as is the common practice for hibernomas. The Soft Tissue Registry of the Armed Forces Institute of Pathology series revealed no recurrence or aggressive behaviour [1]. As hibernomas do not have malignant potential, there is currently no role of neoadjuvant or adjuvant chemotherapy or hormonal therapy. The management should be in line with other benign masses of the breast aligned with principles of breast lumpectomy and reconstruction.

## CONCLUSION

Hibernomas are benign tumours closely related to brown adipose tissue, which is normally present and active in humans from foetal life through adult life. A comprehensive history and triple assessment is the cornerstone to workup and management of these relatively benign masses.

## Data Availability

The data are deemed confidential and under ethics cannot be disseminated openly due to confidentiality and privacy.
